# In science we (should) trust: Expectations and compliance across nine countries during the COVID-19 pandemic

**DOI:** 10.1371/journal.pone.0252892

**Published:** 2021-06-04

**Authors:** Cristina Bicchieri, Enrique Fatas, Abraham Aldama, Andrés Casas, Ishwari Deshpande, Mariagiulia Lauro, Cristina Parilli, Max Spohn, Paula Pereira, Ruiling Wen

**Affiliations:** 1 Center for Social Norms and Behavioral Dynamics, University of Pennsylvania, Philadelphia, PA, United States of America; 2 Management Department, Universidad ICESI, Cali, Colombia; Middlesex University, UNITED KINGDOM

## Abstract

The magnitude and nature of the COVID-19 pandemic prevents public health policies from relying on coercive enforcement. Practicing social distancing, wearing masks and staying at home becomes voluntary and conditional on the behavior of others. We present the results of a large-scale survey experiment in nine countries with representative samples of the population. We find that both empirical expectations (what others do) and normative expectations (what others approve of) play a significant role in compliance, beyond the effect of any other individual or group characteristic. In our vignette experiment, respondents evaluate the likelihood of compliance with social distancing and staying at home of someone similar to them in a hypothetical scenario. When empirical and normative expectations of individuals are high, respondents’ evaluation of the vignette’s character’s compliance likelihood goes up by 55% (relative to the low expectations condition). Similar results are obtained when looking at self-reported compliance among those with high expectations. Our results are moderated by individuals’ trust in government and trust in science. Holding expectations high, the effect of trusting science is substantial and significant in our vignette experiment (22% increase in compliance likelihood), and even larger in self-reported compliance (76% and 127% increase before and after the lockdown). By contrast, trusting the government only generates modest effects. At the aggregate level, the country-level trust in science, and not in government, becomes a strong predictor of compliance.

## Introduction

The COVID-19 pandemic caused by the novel coronavirus SARS-COV-2 has led many countries to implement strict measures to limit the spread of the disease [1]. Advocated by both governments and scientists, these measures go from voluntary social distancing and mask wearing to mandatory stay-at-home policies [2, 3]. Given the scale of the pandemic, the effectiveness of these measures relies on people’s voluntary compliance, as governments cannot coercively enforce them. Governments’ success depends on their legitimacy; the swiftness of their response; how their communication strategy effectively combines rational, moral, and emotional appeals; and their capacity to leverage people’s cognitive biases to effectively influence their behavior [4–14]. Voluntary participation of millions of individuals represents a non-trivial, massive social dilemma, in which individuals may not sufficiently consider the positive externality of compliance. Cooperation in large scale social dilemmas like this one is heavily influenced by the expectations of individuals about the behavior of others [15–17], and the emergence of social norms supporting compliant behavior might significantly increase people’s willingness to comply [18, 19]. Specifically, people’s belief about how others in their reference group are behaving, their *empirical expectations*, and their beliefs about what others believe is the right thing to do, their *normative expectations*, might influence their behavior [19, 20].

In this paper, we analyze how empirical and normative expectations shape voluntary compliance by looking at how individuals condition their behavior to the behavior and normative views of others in a survey experiment run in several countries. More specifically, our survey experiment was run in nine countries in Asia, Europe and the Americas, differentially affected by the pandemic: China, Colombia, Germany, Italy, Mexico, South Korea, Spain, the United Kingdom, and the United States of America. Our study contributes to a growing social scientific literature on the determinants of compliance with behavioral policies set in place to curb the spread of COVID-19. Previous research has studied, for example, the impact of different messaging strategies [9–14], partisanship and ideology [21, 22], expected duration of the policies [23], and misinformation [24] on people’s willingness to comply with the public health measures designed to curb the spread of the disease (for a review see [25]). In particular, our research contributes to a growing literature that emphasizes the role of norms in determining behavior during the pandemic. This literature relies mostly on correlations between perceived norms and self-reported willingness to comply with preventive behaviors [26–29] (though see [10]). Generally, the literature finds a positive correlation between compliance with preventive behaviors and the belief that others comply and approve of compliance (though see [26]). Surveys, however, can be subject to a desirability bias, especially when the questions are about behavior that is perceived as affecting the welfare of society at large. We thus add an experiment in which we present respondents with a vignette describing a hypothetical situation in which we manipulate both the degree at which others comply with the public health measures and the degree at which others believe people should comply with the measures, in a 2x2 factorial design. We randomly assign participants to different conditions, varying empirical and normative expectations about compliance. We present someone similar to the respondent as the protagonist of the vignette, about whom the participant has to assess the likelihood of compliance. Vignettes are important for several reasons. First, when the questions being asked are socially sensitive, a hypothetical scenario provides an unthreatening and impersonal avenue for exploring respondents’ beliefs about a sensitive topic [30, 31]. Second, our hypothetical scenario applies to someone similar to the respondent but not the respondents themselves. One reason is that, for some people it is difficult to imagine themselves in hypothetical or counterfactual situations, since this requires the ability to assume as true claims that may conflict with what is accepted as true. Lack of such ability may lead one to deny that the suggested scenario is possible [20]. Moreover, a social desirability bias might still be at work if the vignette protagonist is still the responder. Finally, vignettes make use of experimental manipulations to arrive at causal knowledge [32]. By varying the empirical and normative expectations of the vignette’s character, we want to assess whether the predicted target behavior is influenced by social expectations. If expectations are causally relevant, the preference to engage in the target behavior will be *conditional* on the social expectations entertained by the agent. In this case, respondents will predict different behaviors (compliance with the rules) depending on the social expectations of the fictitious character [20]. Vignettes also allow us to measure the relative influence of different social expectations, a useful piece of information when we try to enact behavioral change [33].

As a robustness check for the internal validity of the vignette experiment, we compare the experimental outcomes (likelihood of compliance of the vignette character with public health guidelines) with self-reported compliance, both before and after strict enforcement policies are implemented in each country (e.g. a lockdown). To do so, we assess individuals’ self-reported levels of compliance before and after confinement policies were implemented in their countries, as well as their empirical and normative expectations (again, before and after the lockdown).

Our results strongly suggest that increasing both empirical and normative expectations has a substantial and significant impact on people’s willingness to practice social distancing and staying at home. In the survey experiment, the vignette character’s likelihood of compliance with practicing social distancing and willingness to stay at home increases by more than 50%. When only empirical or normative expectations are high (but not both, as in our ‘incongruent’ expectations conditions) there is only a modest increase in compliance likelihood. What are the mechanisms behind this effect? The effectiveness of policies promoting voluntary compliance crucially depends on how individuals perceive these policies and their inner rationale. Institutions trusted by people are typically seen as more credible sources of empirical and normative information [34]. The multidimensional nature of the pandemic may require not only trust in the agent (e.g. a government agency delivering a compliance message), but also trust in the messenger providing its underlying logic (e.g. the scientific rationale for confinement, wearing masks, or social distancing). The uncertainty created by infectious outbreaks [35] represents a serious challenge to any policy promoting voluntary compliance, as beyond the level of trust in the agent (e.g. the government) recipients of any message must also be convinced by the trustworthiness of the messenger (e.g. epidemiologists, virologists, scientists in general) [36]. Unfortunately, since the pandemic started, the messages of scientists and the messages of governments have not always been consistent [37].

We hypothesize that the degree to which people practice social distancing and stay at home depends on perceived social norms (as defined by the joint effect of empirical and normative expectations). However, we recognize that compliance might be ultimately driven by their trust in the institutions and people that promote or back up compliant behaviors. In other words, we are interested in learning whether empirical and normative expectations affect people’s willingness to comply with social distancing and staying at home policies, but also whether trust in scientists and government moderate this relationship [7, 36, 38]. In particular, we theorize that if people do not trust the institutions that promote public health behaviors, the effect of social norms on compliance with the behaviors may be dampened. When both expectations and trust in institutions that promote behaviors to mitigate the spread of COVID-19 are low, people may assess the risk of contagion as particularly low. However, when expectations are high, people that do not trust these institutions may still be tempted to free ride if they believe other people are complying.

Thus, we explore how the individual level of trust in different institutions and groups shape compliance, and interact with high empirical and normative expectations. We specifically focus on two groups: government and science. The logic behind this focus is straightforward. The lack of trust in government may lower the credibility of government policies and generate low compliance with public health guidelines [39, 40]. The lack of trust in science may actually decrease the expected effectiveness of public health policies. For example, in the US, after lockdown policies were implemented an additional 5 percent of people stayed home in counties with above median belief in anthropogenic climate change compared to those with below median belief in climate change as measured with cell phone mobility data [38]. Given the often-confusing messages towards the validity of scientific evidence sent by governments during the COVID-19 pandemic, we find the analysis of this trade-off extremely relevant to actual policies.

Our results strongly suggest that both types of trust play very different roles. Generally, high levels of trust in government generate modest increases in compliance likelihood (relative to the level of compliance observed among those with low levels of trust in government). As respondents in our survey experiment are randomly assigned to one of four conditions, we can cleanly disentangle the effect of expectations from the effect of trust. High trust in governments plays a modest effect when expectations of compliance are high in our vignette experiment. Consistent with previous results [41, 42], when individuals have high trust in government, likelihood of compliance with the public health guidelines (social distancing and staying at home) goes up by a modest amount (relative to those with low trust in government). In sharp contrast, in the same high expectations condition, a high level of trust in science generates a much larger increase in likelihood of compliance. Interestingly, we obtain very similar results when studying the effect of trust in government and trust in science on self-reported compliance.

Our results document how trust in science is a necessary means to achieve compliance, even in the most favorable scenario (with high empirical and normative expectations about others’ compliance), and regardless of the level of trust in government. Capping trust in science by challenging the legitimacy and rigor of scientific evidence may come at a very large price: a major and pernicious effect on compliance, facilitating the spread of the pandemic. In the rest of the paper, we describe the survey in section 2, present the results in section 3 and discuss the limitations and policy lessons of our study in the final section.

## Materials and methods

The study was approved by the University of Pennsylvania IRB under protocol number 843052. Consent was obtained in written form before the beginning of the survey. Our survey was administered in China, Colombia, Germany, Italy, Mexico, South Korea, Spain, the United Kingdom, and the United States between late April and early May of 2020. It was translated and back translated into the language spoken in each of the countries by native local speakers, with a national of each country being in charge of the version run in each country (Colombian, Mexican and Spanish nationals were in charge of the versions administered in Colombia, Mexico and Spain, and the same version was administered in the USA and the United Kingdom, as the only exception). The survey was administered to samples of around 1,250 respondents per country through panels of respondents previously recruited by survey companies. Representative samples were balanced in terms of age and gender. Demographic characteristics are reported in S1 Table. Before answering the survey, potential respondents were informed about the nature and duration of the study and asked to provide consent.

Our survey focuses on compliance with two specific policy guidelines, common across the nine countries studied (social distancing and staying at home, described to participants in plain words, see the instructions in the Supporting Information). Our method is grounded on the theory of social norms [19] and follows its standard methodology [20]. Our first research question is to determine whether individual choices to comply are affected by social expectations. We consider a social norm to be in place whenever people choose to comply with an observable (but not necessarily official) rule of behavior not just because it is consistent with their personally held normative beliefs, but also because their expectations about others support it. Whenever social norms are at play, an individual’s decision to conform with a rule of behavior is conditional on their first-order beliefs about whether people in a reference group follow that rule (*empirical expectations*) and on their second-order beliefs about whether they think that others believe one ought to obey the rule (*normative expectations*).

The survey was administered online, divided in 8 blocks (see S1 Fig), and took around 7 minutes to complete. For the purpose of this paper, the survey has four components of interest. In blocks 1 and 2 (questions 1 to 14), we elicit information about the individual characteristics of participants, including attitudes towards, exposure to, and risk assessment of the pandemic (see S1 Table). In blocks 3 to 6 (questions 15 to 30), we elicit their individual degree of compliance with the recommended practices, their personal normative beliefs, and their empirical and normative expectations, before and after the enforcement of strict compliance rules, linked to the moment in which countries imposed lockdowns. In block 8 (questions 33 and 34, with multiple entries) we measure their level of trust in different groups (see S2 and S3 Tables).

The core of the survey is the vignette experiment in which we study whether likelihood of compliance is conditional on expectations. The vignettes used in the survey experiment were simple. Individuals were asked to imagine a specific situation and then were asked to answer two simple questions about the vignette protagonist’s level of compliance. The situation was described in clear terms: “Somebody like you lives in a very similar country that is affected by Coronavirus (COVID-19). *Most*/*Few* residents are practicing social distancing and staying at home apart from unavoidable and necessary trips. *Most/Few* residents also believe that one should practice social distancing and stay at home apart from unavoidable and necessary trips”, with the manipulation in italics. As empirical and normative expectations could be either High (Most) or Low (Few), our design follows a 2x2 factorial design, with congruent expectations in two conditions (High/High or Low/Low), and incongruent expectations in the other two (High Normative and Low Empirical or Low Normative and High Empirical). After the description of the situation, participants were asked to answer two simple questions on a 1–10 scale (our measures of likelihood of compliance): “How likely is this person to practice social distancing/stay at home in this situation? Subjects were randomly assigned to one condition, following a between-subjects protocol.

Our methodology has several merits. First, it gives us the chance to claim causality from the random assignment to one, and only one, condition. If predicted compliance is significantly different between low and high (congruent) expectations, we can conclude that preferences for compliance are conditional on expectations. Incongruent expectations (low normative and high empirical or vice versa) are interesting as they can tell us if some expectations are more important than others in driving behavior. However, in this case we did not focus on this issue, since, though incongruent expectations lead to better outcomes than congruent low expectations, their effect is muted compared to the effect of increasing both normative and empirical expectations at the same time. Second, an advantage of using a hypothetical situation with a fictitious character is that it moderates demand effects that are common with direct surveys or even with vignettes that measure the respondents’ behavioral intentions in response to a hypothetical situation. Third, all respondents were asked in blocks 3 to 6 about their actual level of compliance with the public health guidelines -our measure of self-reported compliance- (“did you/are you practicing social distancing/stay at home apart from unavoidable and necessary trips?”). Respondents also answered a question about their personal normative beliefs (“did/do you believe that one should practice social distancing/staying at home?”). They were also asked about their empirical and normative expectations (“estimate the percentage of fellow residents who were/are practicing social distancing/staying at home before/since the countrywide Coronavirus (COVID-19) lockdown” and “the percentage of fellow residents who believed/believe that one should practice social distancing/stay at home apart from unavoidable and necessary trips before/since the countrywide Coronavirus (COVID-19) lockdown”). This allows us to compare the outcome of the survey experiment (likelihood of compliance) with the levels of compliance as self-reported by the same individuals in blocks 3 to 6. As we elicit the empirical and normative expectations of all individuals, we can also check how reliable the link between expectations (exogenously imposed in the vignette experiment) with individuals holding the same expectations (endogenously reported by them). This comparison serves as a robustness check. As individuals are randomly assigned to one of four conditions in the vignette experiment, we believe it is hard to associate any result with the rationalization of previous responses.

In order to assess the interaction between trust and compliance, we asked participants about their general level of trust (using the binary question used by the World Value Survey) and about their individual level of trust in people from different groups, using a 1–4 scale (from “Not at all” to “Completely”). The list of groups includes scientists, the government, and other categories (including families, neighbors, the police, and medical doctors). We will concentrate on trust in the government and scientists. For the sake of exposition, we will be transforming the 1–4 scale into a binary one, with levels 1–2 associated with low levels of trust (Low trust takes the value of 0) and levels 3–4 to high levels of trust (High trust takes the value of 1). Similar, almost identical, results are obtained when using the 1–4 scale. Descriptive statistics and correlations for measures of trust are in S2 and S3 Tables.

## Results

In the main text, we focus on results at the aggregate level but present results by country in the Supporting Information. Our analysis strategy relies on pairwise comparisons to compare compliance likelihood between conditions. We run non-parametric tests of compliance likelihood (as observed in the different conditions of the vignette experiment) and self-reported compliance (as elicited in blocks 3 to 6, see S1 Fig, for different expectations). Given that compliance likelihood is collected using a Likert scale and self-reported compliance is elicited in a binary survey question (yes or no), the tests used in each comparison are described in detail in every table. We will then compare compliance likelihood and self-reported compliance for different levels of trust in government and in science using also non-parametric tests. We assess the effect of the manipulations on compliance likelihood with normative and empirical expectations and trust in government and science as predictors, controlling for country fixed effects and individual characteristics in the supplementary materials, even when we refer to the robustness checks in the main text.

Table 1 presents data on behavior, normative beliefs and empirical and normative expectations, before and after the lockdown was introduced in each country. At the aggregate level (n = 11,558), self-reported compliance before the lockdown (73% and 74% with social distancing –SD– and staying at home –SH–, respectively) is high but consistent with the proportion of participants supporting the safety measures (76% and 73%). Empirical expectations about others practicing social distancing and staying at home (45% and 45%) and normative expectations about people’s normative beliefs (47% and 46%) indicate that most participants think others to be far less compliant or convinced about the necessity of the measures than themselves (although we cannot discard the possibility that people’s response to the question about their own normative beliefs is driven by social desirability). S4 Table shows the same data at the country level.

**Table 1:** Behavior, normative beliefs and expectations.

	Social distance (SD)	Stay home (SH)
Before the lockdown		
Behavior (%)	0.74	0.73
Normative belief (%)	0.76	0.73
Empirical expectation (0–100)	44.76	44.99
	(28.58)	(29.42)
Normative expectation (0–100)	46.51	45.79
	(29.84)	(30.09)
After the lockdown		
Behavior (%)	0.97	0.95
Normative belief (%)	0.96	0.94
Empirical expectation (0–100)	70.46	69.75
	(22.72)	(22.94)
Normative expectation (0–100)	72.07	71.17
	(22.72)	(23.04)
Change (%)		
Behavior	0.22	0.23
Normative belief	0.20	0.22

Note: Standard deviations in parentheses.

After the lockdown, the self-reported measures for individual behaviors and support for the safety measures are very high (95% and 97% compliance, 96% and 94% normative beliefs). Previous studies using cell phone location data in the US suggest that the introduction of lockdowns only increased the proportion of people staying at home by about 5% [43]; thus, we cannot discard a bias in the way respondents answered these questions. Empirical and normative expectations are still well below individual behaviors and normative beliefs (70% and 72% for social distancing, 70% and 71% for staying at home). S5 Table presents the same data at the country level. Compliance with the two prescribed behaviors are highly correlated (correlation coefficients at the country level between .38 and .75 before the lockdown and between .28 and .63 after the lockdown), and personal normative beliefs are strongly and positively correlated (between .48 and .74 before, between .29 and .64 after). Overall, only 19% of our respondents complied with one behavior but not the other before the lockdown (13% after the lockdown).

The introduction of a lockdown caused more than a fifth of all participants to report a shift in their behavior and beliefs towards compliance, as presented in the bottom part of Table 1. This proportion significantly varies across countries, which follows from the very different starting level of compliance (e.g. only 9% more people practice social distancing in South Korea after the lockdown, versus 34% in Spain). Changes in normative beliefs also vary in magnitude (9% increase in favor of staying at home in Mexico, versus 34% in the UK). A majority of respondents reacted strongly and positively to the introduction of lockdown adapting their normative and empirical expectations (see S3 Fig, with the proportion of individuals improving their expectations).

Our experimental data are presented in Table 2. We present a similar analysis at the country level in S6 Table. The likelihood of compliance with the two prescribed behaviors are highly correlated (correlation coefficients at the country level are between .81 and .92). When asked to indicate the vignette character’s likelihood of compliance with social distancing and staying at home on a 1–10 Likert scale, our pool strongly conditions behavior on the expectations described in the vignette. There are substantial differences between congruent social expectations (High Normative–High Empirical or Low Normative–Low Empirical) and minor ones between incongruent ones (High Normative–Low Empirical, Low Normative–High Empirical). While compliance goes up by 55% in High–High relative to Low–Low (and of a similar magnitude in the nine countries studied), differences between incongruent vignettes is only 5% (and not systematic across the nine countries, as the erratic path of ordering in S6 Table shows). S7 Table shows the same results in a linear regression including demographic and country controls and corroborates the above findings.

**Table 2:** Experimental outcomes.

	High/High	High/Low	Low/High	Low/Low
Stay home	7.57	6.06	5.77	4.93
	(2.34)	(2.67)	(2.70)	(2.95)
Social distancing	7.63	6.08	5.78	4.88
	(2.37)	(2.65)	(2.71)	(2.94)

Note: Standard deviations in parentheses. High/High refers to both normative and empirical expectations being high; High/Low refers to High normative expectations and Low empirical expectations; Low/High refers to Low normative expectations and high empirical expectations; and Low/Low refers to both normative and empirical expectations being low.

**Fig 1:**
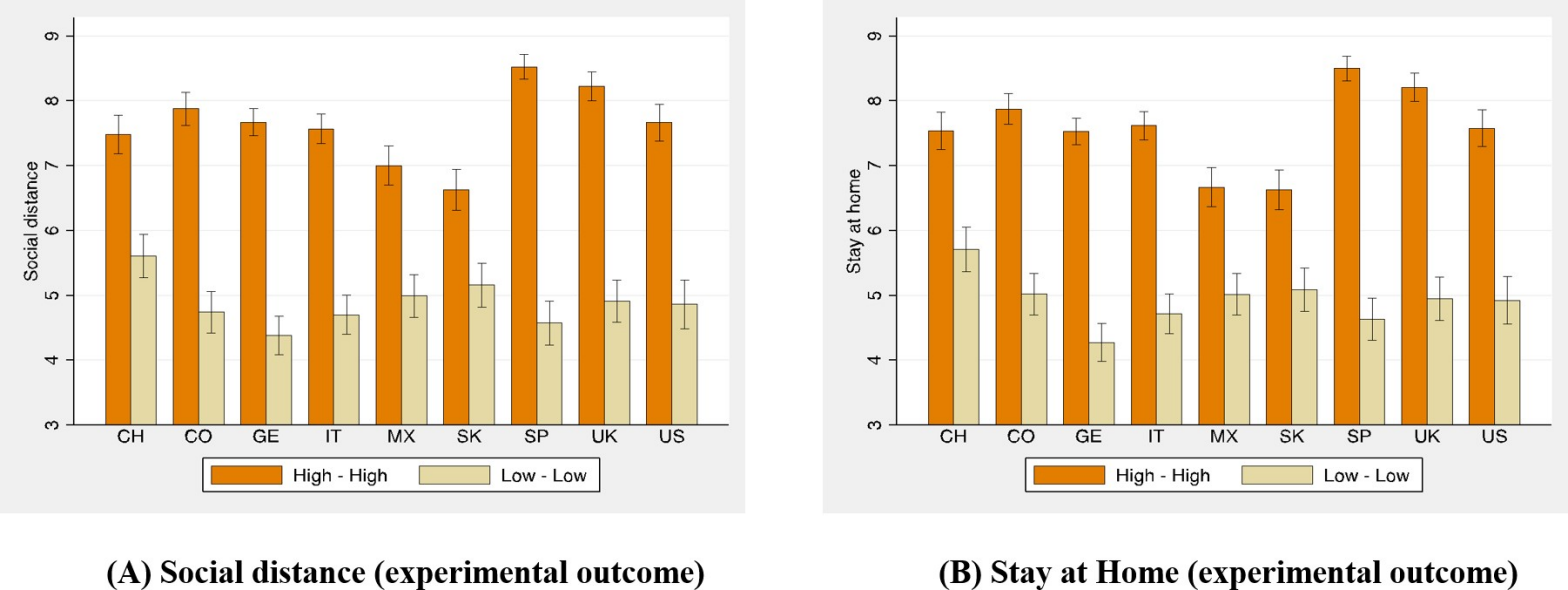
The new social norms.

Fig 1 compares the compliance decisions made by our participants in the hypothetical vignette in congruent conditions (High Normative-High Empirical–or High-High–and Low Normative-Low Empirical–or Low-Low), showing how they condition compliance decisions on the empirical and normative expectations of the vignette (S2 Fig presents the same comparison across the four experimental conditions). In each country, every single pairwise comparison of compliance likelihood assessed when expectations are High-High as opposed to when expectations are Low-Low is significant at the p < .001 level (using a Mann-Whitney test) and large, from the 29% (30%) compliance increase in social distancing (and staying at home) in South Korea to the 86% (83%) compliance increase in Spain. Compliance likelihood is also significantly higher in the High-High condition than in the incongruent treatments (either High-Low or Low-High), with one exception (High-High and High-Low are not significantly different in South Korea, p = 0.6461 and p = 0.2018 for Social Distance and Stay at Home). Similar pairwise comparisons between incongruent expectations yields a mixed, nonsystematic picture. While compliance likelihood is significantly higher in High-Low than in Low-High in Mexico and South Korea (for both Social Distance and Stay at Home, p = 0.0087, p<0.0000, p = 0.0240 and p<0.000, respectively), it is significantly lower in Germany (p = 0.0029 and 0.0481, respectively) and follows a nonsystematic pattern in the other countries (no significant differences in Spain and the United Kingdom, marginally larger in High-Low than in Low-High in the US, p = 0.0962 and 0.0531, and Italy, p = 0.0346 and 0.0582, and marginally lower in China, p = 0.0485 and p = 0.0885, respectively).

**Fig 2:**
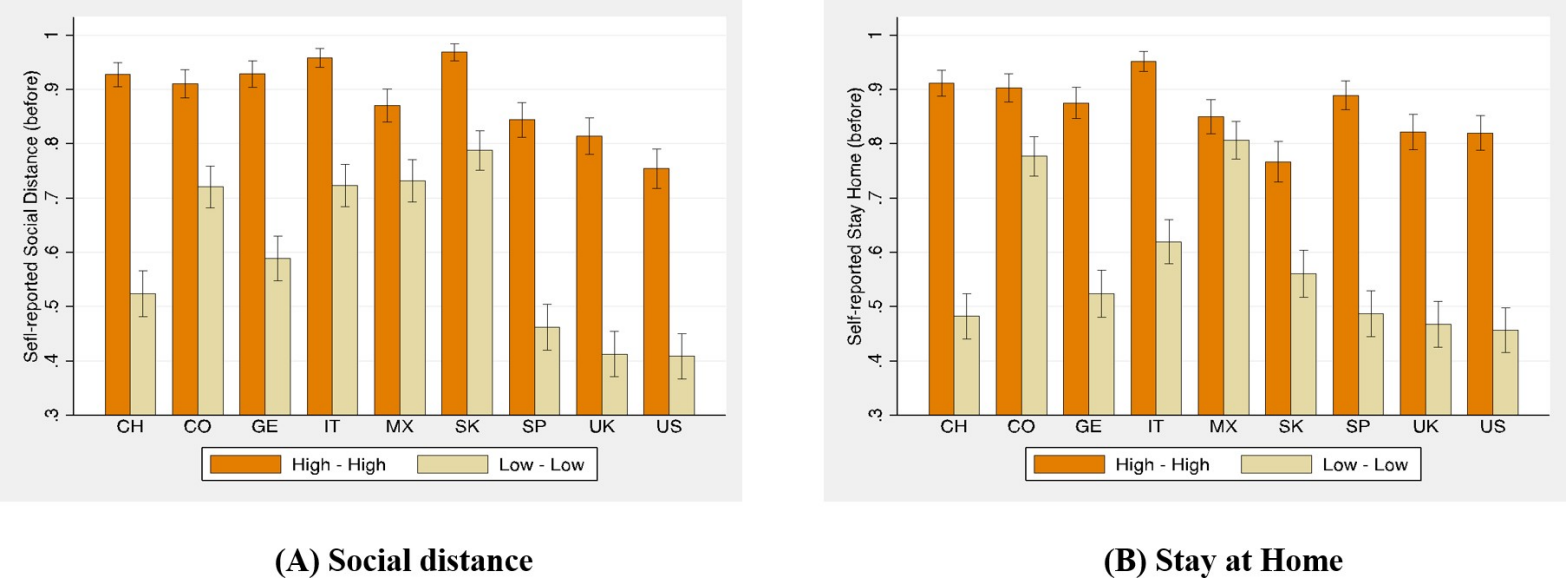
Self-reported compliance and expectations.

As Fig 1 captures compliance in a hypothetical scenario, in which respondents are asked to imagine a similar country to the one they live in, suffering similarly from the COVID-19 pandemic, we run a simple robustness check. As noted above, participants in our survey experiment also self-reported their level of compliance before and after the lockdown. We look at how participants in our survey experiment condition their self-reported compliance on their expectations in Fig 2. We split each country’s sample by their level of expectations. Those participants with high empirical expectations (above the country median) and high normative expectations (again, above the country median) share the optimistic expectations of all participants in the High–High condition in the vignette experiment. Participants below the median empirical and normative expectations are our control group for the Low–Low condition.

While expectations are exogenously and randomly imposed on participants in each condition in the vignette experiment, expectations are endogenous when self-reporting compliance before and after the lockdown. Moreover, all participants self-report their compliance first, and then are randomly assigned to one of the four conditions in the vignette experiment, so rationalization of previous decisions cannot explain much. Fig 2 presents the mean compliance before the lockdown, and binomial confidence intervals, of those participants with optimistic expectations (High–High) and pessimistic expectations (Low–Low), leaving aside those holding incongruent expectations (High–Low or Low–High, see S3 Fig). Fig 2 generates a very similar message to the one observed in Fig 1. In every country, with the partial exception of Mexico, participants with higher expectations self-report higher levels of compliance, and the comparison is statistically significant at the p < .001 level (using two-sided Fisher test, p-values are always below 0.05, with the exception of Mexico when self-reporting staying at home, p = 0.077).

**Fig 3:**
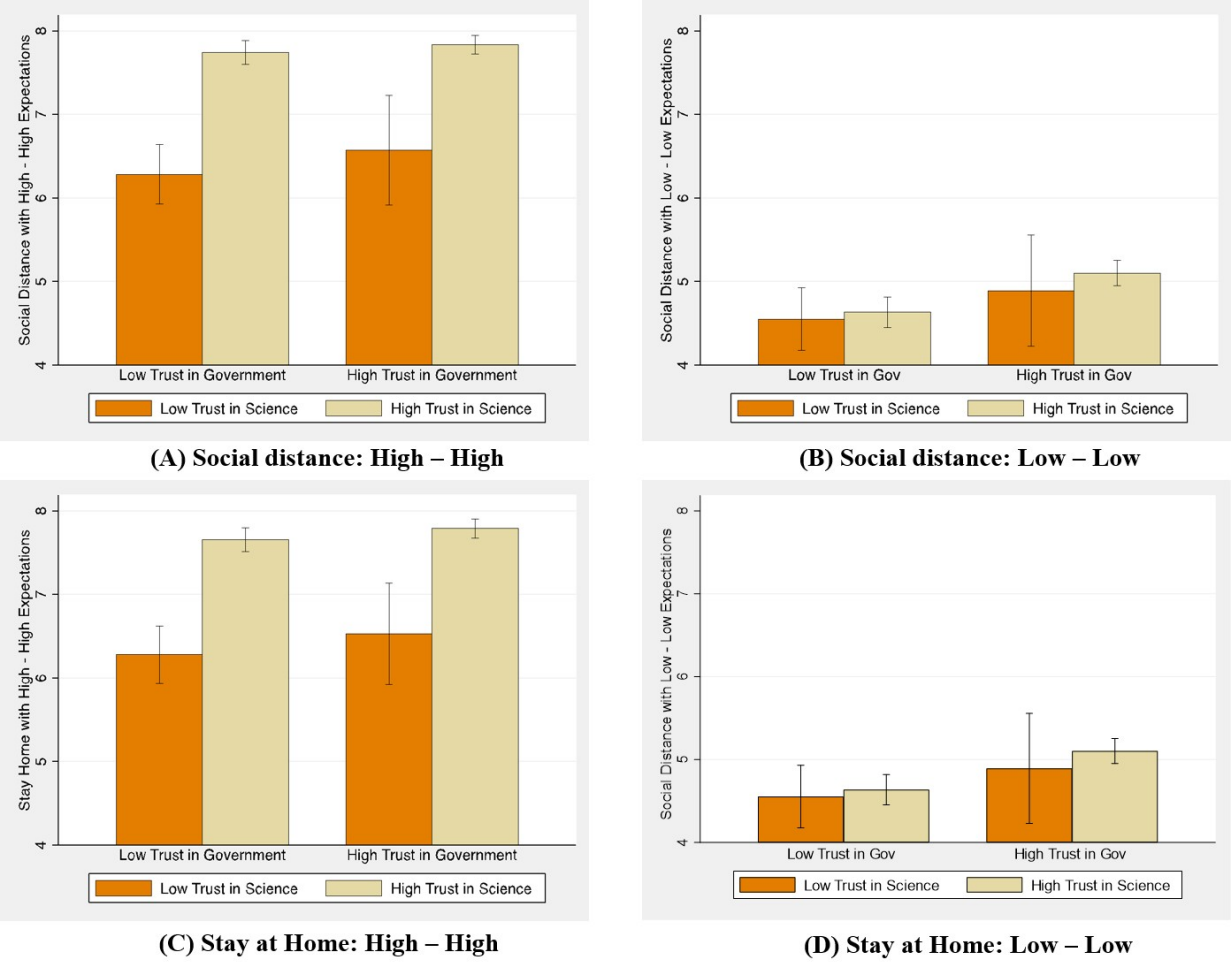
Social distance compliance and trust.

What is the mechanism behind the strong connection between compliance and expectations? Fig 3 shows the link between trust in different groups (science and government) and compliance plotting compliance in the vignette experiment, when both expectations are high (High Normative and High Empirical expectations), with bars representing the mean level of compliance and 95% confidence interval added to each bar to facilitate the analysis. In Fig 3, dark orange bars measure compliance likelihood for those individuals holding low levels of trust in science, light khaki bars compliance for those individuals holding high levels of trust in science. In each figure, different bars are created for individuals with low and high trust in government.

We interpret Fig 3 by measuring the impact of trust on compliance likelihood. In Fig 3A and 3C, with high congruent expectations, compliance likelihood is maximized when trust in science is high (every comparison between the dark orange bar and its corresponding light khaki bar is significant at the 1% level using a Mann-Whitney test). Compliance likelihood among those holding high levels of trust in government is not significantly higher than compliance likelihood among those with low levels of trust in government, regardless of their level of trust in science (if high, p-value = 0.3460, if low p-value = 0.3596 for social distance; p-value = 0.4958 and p-value = 0.1778 for stay at home, using the same non-parametric test).

When both expectations in the vignette experiment are low, Fig 3B and 3D show a dramatically different picture. Those holding high and low levels of trust in science exhibit very similar levels of compliance likelihood when both normative and empirical expectations are low (p-values are 0.6305, 0.5761, 0.7815 and 0.2753 for high and low levels of trust in government, and social distance and staying at home). When expectations are low, compliance likelihood is significantly higher among those with high levels of trust in government only when trust in science is also high (p-values 0.0001 and 0.0000 for social distance and staying at home), but not when trust in science is low (p-values are 0.3313 and 0.7892). Even when compliance likelihood among participants with low trust in science and in government is still high, we refrain from speculating about the interpretation of absolute levels and focus on comparative statics.

Table 3 summarizes our results for the experiment. It shows that the largest increase in compliance likelihood with both social distancing and staying at home occurs in respondents in the High-High condition. Those who have a high trust in science are 23 percent more likely to comply with social distancing measures and 22 percent more likely to comply with staying at home measures than those with low trust in science.

**Table 3:** Compliance likelihood and trust in science and government.

	Trust in Science			Trust in Government		
Social distance	Low		High	Low		High
Low-Low	4.63 (2.97)	*+6%*	4.91 (2.93)	4.62 (2.93)	+10%***	5.09 (2.92)
High-High	6.35 (2.88)	+23%***	7.80 (2.24)	7.44 (2.47)	+4%***	7.77 (2.28)
Stay at home	Low		High	Low		High
Low-Low	4.71 (3.00)	*+5%*	4.96 (2.94)	4.64 (2.90)	+11%***	5.16 (2.97)
High-High	6.34 (2.76)	+22%***	7.74 (2.22)	7.37 (2.44)	+5%***	7.73(2.24)

High trust in Science (Government) if trust in Science is “Completely” or “Somewhat”, Low trust in Science (Government) if Trust in Science (scientists) is “Not very much” or “Not at all”. Low-Low (High-High) corresponds with the questions in the vignette experiment in which Most residents practiced Social distance or Stay at home and Most residents believed that everybody should practice it. Standard deviations are between parentheses.

*** p-value<0.01

** p-value<0.05

* p-value<0.1 from Mann-Whitney tests

S8 Table shows these results in a linear regression controlling for individual covariates and country fixed effects. As a robustness check, S9 Table also presents the results of a linear regression that controls for individual covariates and country fixed effects, but using the raw 1–4 trust scale as a moderator. Both tables show the same results: the effect of High-High expectations on compliance likelihood increases as trust in science increases but not as trust in government increases.

**Fig 4:**
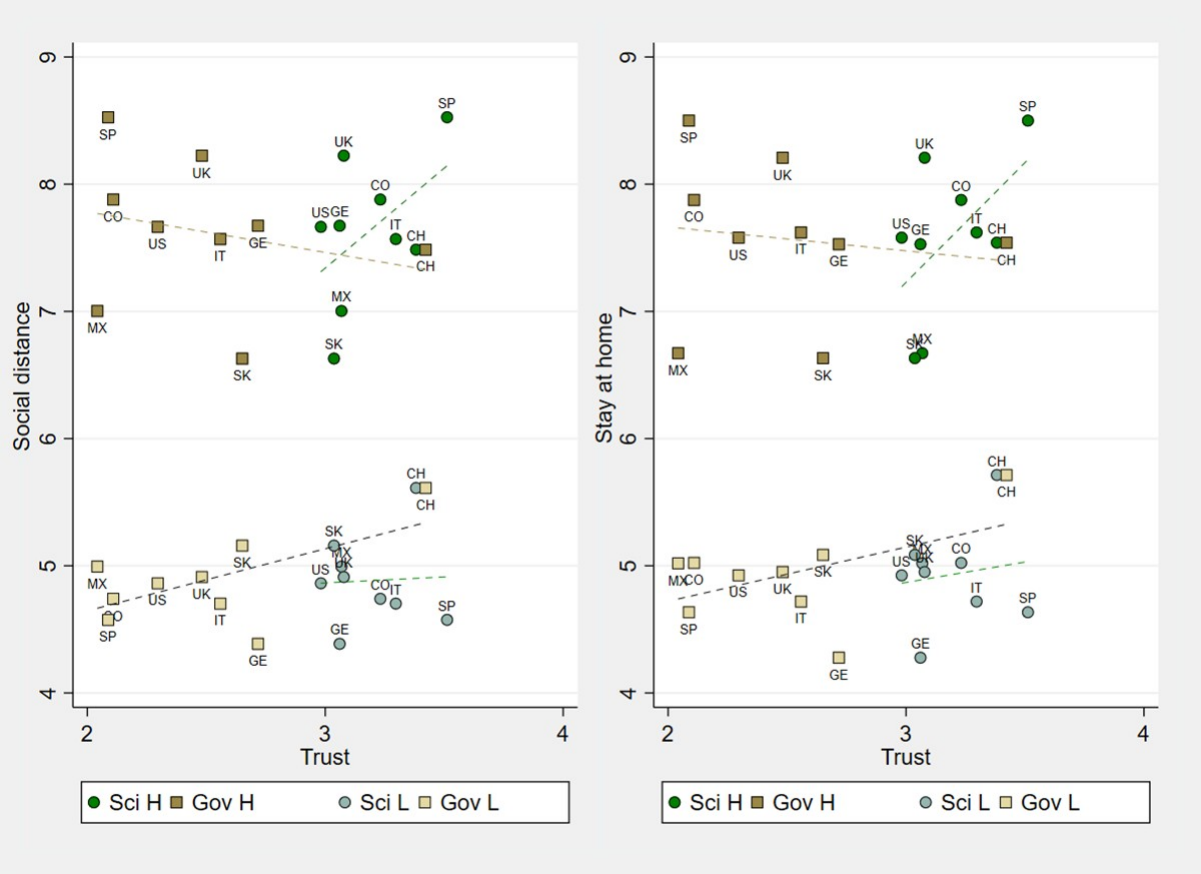
Country level compliance likelihood and trust in science and government. Compliance likelihood at the country level (mean) for High (H) and Low (L) expectations (as in treatments High-High and Low-Low, respectively) and Trust in Science and Government using the 1–4 scale.

Fig 4 ranks countries by their level of trust in science and trust in government and plots the country’s average compliance likelihood for social distance and staying at home. Fig 4 confirms the results of our previous analysis. When both normative and empirical expectations are high, the country level correlation between compliance likelihood and trust in science is strong and positive, but weakly negative for trust in government. When expectations are low, we observe a positive correlation between trust in government and compliance and no correlation for trust in science.

We again check whether self-reported compliance follows the pattern described above in Table 4, showing the proportion of participants with self-reported full compliance (both practicing social distance and staying at home) by their level of trust in science and government, when both expectations are either high or low (leaving aside those with incongruent expectations). Before the lockdown, when expectations are low, compliance does not improve when comparing participants with low and high levels of trust in science or government (it actually goes down by 9% and 7%). However, when expectations are high, high trust in science increases compliance by 76% (and high trust in government only by 29%). This big jump is not only significant using a Pearson chi-squared test (p-value<0.0001 in both cases), but large, and in line with the results observed in the vignette experiment. A similar result can be obtained when looking at compliance levels after the lockdown: modest effects with low expectations (13% increase for high level of trust in science, and 2% for high levels of trust in the government) and large and very different effects when expectations are high (+127% for trust in Science and +36% for trust in government). S11 Table confirms this result by reporting the marginal effects of a probit estimation with individual and country controls.

**Table 4:** Self-reported compliance and trust.

	Trust in Science			Trust in Government		
Before	Low		High	Low		High
Low Exp	0.4980	-9%*	0.4536	0.4782	-7%**	0.4427
High Exp	0.4704	+76%***	0.8301	0.6564	+29%***	0.8437
After	Low		High	Low		High
Low Exp	0.8161	+13%***	0.9205	0.8956	+2%*	0.9119
High Exp	0.4331	+127%***	0.9811	0.7253	+36%***	0.9846

High trust in Science (Government) if trust in Science is “Completely” or “Somewhat”, Low trust in Science (Government) if Trust in Science (scientists) is “Not very much” or “Not at all”. Participants qualify as Low Exp (High Exp) if their empirical and normative expectations are below (above) the country median.

*** p-value<0.01

** p-value<0.05

* p-value<0.1 from Chi Square tests.

Table 4 shows a clean and policy relevant result: destroying trust in science comes at a very high cost, as even holding high empirical and normative expectations, participants with low levels of trust in science exhibit a very poor level of compliance, as only 43% of them fully comply with the public health guidelines.

## Discussion and limitations

A growing literature analyzes the role of a host of factors that affect compliance with public health measures designed to curb the spread of COVID-19 [25]. We focus only on one of these factors, the role of norms and how their effect is moderated by trust in science. A main result of our study is that trust in science is needed to achieve the maximum level of compliance. In this respect, it is interesting to compare the effects of trust in science for individuals who hold high versus low expectations. An individual with high expectations of others’ compliance will be tempted to free ride, especially if there is doubt about the rationale, and plausibility, of the restrictive measures. The two forces of doubt and the temptation to free ride converge to encourage non-compliance. As the risk of infection is low and the seriousness of the disease is in doubt, the cost of compliance becomes quite high. For people holding low expectations, there is no incentive to free ride, as it is believed that most people do not comply with the policy recommendations. There is, however, a risk in not taking precautions, since infected people are moving around freely. Doubts about science in this case play a minimal role in determining behavior. If the probability of contracting the viral infection is high, even if the seriousness of the disease is in doubt, compliance should not be too costly.

Our results notably show that having high expectations of compliance is not enough to induce further compliance. This is an important consideration when using norm nudging to foster prosocial behavior. Norm nudging works by providing information about what similarly situated people do or approve of. The assumption is that inducing adequate social expectations will change behavior in a prosocial direction [44]. In the case of a public health threat, norm-nudging effects could be nullified by mistrust in science. Norm nudging in this case could potentially work only if accompanied by information that strengthens confidence in the results of scientific research. In particular, it is important that governments and scientists do not send discordant information, creating uncertainty and skepticism about the right measures to adopt.

A further question is whether trust in government plays a role in increasing compliance with public recommendations. In line with recent work on the legitimacy of public policies [45], when expectations of compliance are high, trust in government is irrelevant, what really matters is only trust in science. When expectations are low, trust in government has only secondary importance. Our results dovetail those of Battiston et al. [46], who argue that trust in science and experts increases both people’s knowledge about the pandemic and their willingness to update their misperceptions with respect to the virus when new information is presented. As a new norm emerges only those who, influenced by scientific research, are willing to change their behavior will comply with the new norm. This highlights the importance of fostering trust in science, rather than just fostering trust in government, in order to ensure the effectiveness of norm nudging in increasing compliance with public health recommendations.

Of course, our research has some limitations. Though vignettes have been shown to approximate real world behavior [47], some studies show divergence between hypothesized behaviors and real world behaviors [48]. Even if the internal validity of the vignette experiment is ensured by randomly assigning respondents to one of the four vignette versions and controlling for covariates, external validity may be questioned. Most research using vignettes, however, measures the reported behavioral *intentions* of the respondents themselves against their realized behavior in similar real life situations to those described in the vignette. Especially in norm-relevant situations, social desirability bias may play a role in hampering external validity, as the vignette is likely to measure responders’ views about how one *should* act in the situation, not how one will in fact act. When the subject of the vignette is a third party instead, the respondents’ assessment of what others would do may provide more accurate responses, as answering questions about others’ behavior attenuates social desirability bias. A further potential difficulty is construct validity, i.e., whether the situation described in the vignette approximates a real life situation the respondent may encounter. To mitigate this problem, our vignette intentionally asks the respondent to imagine someone in a similar situation, leaving the respondent free to fill in the relevant details.

Though our results on the interaction between perceived norms and trust in science also hold for self-reported behaviors, it is possible that some social desirability remains. However, given that a number of studies suggest that both trust in science and expectations matter for determining compliance with behaviors that mitigate the spread of COVID-19, we are confident of the validity of our approach. It is important to note in this respect that, though trust in science may (and usually should) influence personal normative beliefs, it may not influence expectations (and thus social norms), unless one believes that most others, too, trust science. Since norm-compliant behavior largely depends on social expectations, trust in science can only act as a moderator, not as an enabler of these expectations.

Finally, our research only offers a snapshot of the pandemic. People’s expectations about the behavior of others and trust in science may have evolved in different ways in different countries, particularly given the variation in success of mitigation efforts. Further research, using panel data, may address how norms, trust in science, and compliance with public health behaviors have changed throughout the pandemic.

## Supporting information

S1 Table Demographics and coronavirus attitudes and exposure. (PDF)

S2 Table Trust–country level. All measures are on a 1–4 scale, where 1 is the lowest level of trust and 4 is the highest, except the Trust question that is used in the World Values Survey, which takes a value of 1 if people say they can generally trust others and 0 if not.
(PDF)

S3 Table Pairwise correlations between trust in different groups. (PDF)

S4 Table Before the lockdown–country level. (PDF)

S5 Table After the lockdown–country level. (PDF)

S6 Table Experimental outcomes–country level. (PDF)

S7 Table Compliance likelihood (SD and SH) and expectations. Standard errors in parentheses, ** p<0.01, * p<0.05. OLS estimates with individual (gender, age, education and location) and country controls. Low-Low treatment used as the benchmark
(PDF)

S8 Table Trust in science and government (High), compliance likelihood (SD and SH) and expectations. Standard errors in parentheses, ** p<0.01, * p<0.05. OLS estimates with individual (gender, age, education and location) and country controls. Low-Low treatment used as the benchmark.
(PDF)

S9 Table Trust in science and in government (1–4 scale), expectations, and compliance likelihood (SD and SH). In columns 1 and 2 trust (1–4) refers to the level of trust in science; in columns 3 and 4 it refers to having the level of trust in government. Standard errors in parentheses, ** p<0.01, * p<0.05. OLS estimates with individual (gender, age, education and location) and country controls. Low-Low treatment used as the benchmark.
(PDF)

S10 Table Trust in government and science (High) interaction by different levels of expectations and compliance likelihood (SD). Standard errors in parentheses, ** p<0.01, * p<0.05. OLS estimates with individual (gender, age, education and location) and country controls.
(PDF)

S11 Table Probit results for self-reported compliance. Probit estimations with individual (gender, age, education and location) and country controls, figures are marginal effects, relative to the benchmark (low expectations and low level of trust), using self-reported compliance before the lockdown as dependent variable.
(PDF)

S1 Fig Survey sequence. (TIF)

S2 Fig The new social norms. (TIF)

S3 Fig Self-reported compliance. Per the survey, not including incongruent expectations.
(TIF)
