# Supplementary material for: In science we (should) trust: Expectations and compliance across nine countries during the COVID-19 pandemic
Source: PLoS One. 2021 Jun 4;16(6):e0252892. doi: 10.1371/journal.pone.0252892 (PMC8177647; doi:10.1371/journal.pone.0252892)
Supplement: S1 Table — (PDF) [file pone.0252892.s001.pdf]

S1 Table. Demographics and coronavirus attitudes and exposure

	CH	CO	GE	IT	MX	SK	SP	UK	US
Observations	1,239	1,286	1,253	1,271	1,377	1,218	1,259	1,294	1,361
Female (%)	0.47	0.51	0.50	0.51	0.52	0.48	0.49	0.51	0.49
Urban (%)	0.66	0.88	0.31	0.38	0.74	0.56	0.75	0.29	0.38
Age	39.27	40.75	44.65	49.80	34.90	41.28	45.67	49.36	46.82
	(12.20)	(16.49)	(16.22)	(16.39)	(13.50)	(16.93)	(16.02)	(17.95)	(17.66)
Income (1-9)	8.05	4.11	5.74	5.00	5.78	6.58	5.39	4.87	5.79
	(2.06)	(2.31)	(2.22)	(2.29)	(2.86)	(2.75)	(2.33)	(2.33)	(2.83)
Exposure	0.20	0.11	0.18	0.31	0.11	0.12	0.59	0.24	0.30
	(0.40)	(0.32)	(0.39)	(0.46)	(0.31)	(0.33)	(0.49)	(0.42)	(0.46)
Relocated	0.06	0.00	0.01	0.02	0.03	0.09	0.02	0.04	0.14
	(0.24)	(0.07)	(0.12)	(0.13)	(0.16)	(0.28)	(0.13)	(0.20)	(0.34)
Likely individual is infected (1-11)	3.98	4.09	5.32	4.34	3.87	3.66	5.59	5.25	4.99
	(2.78)	(2.56)	(2.52)	(2.46)	(2.62)	(2.72)	(2.51)	(2.50)	(3.00)
Likely family is infected (1-11)	4.17	4.66	5.64	4.49	4.24	3.93	6.23	5.81	5.36
	(2.86)	(2.69)	(2.55)	(2.53)	(2.67)	(2.66)	(2.57)	(2.64)	(3.01)
Likely avg, person is infected (1-11)	6.09	7.04	6.45	6.93	5.71	4.67	7.56	6.38	6.19
	(3.01)	(2.33)	(2.24)	(2.26)	(3.13)	(2.78)	(2.02)	(2.13)	(2.59)
Optimistic bias	0.67	0.82	0.55	0.77	0.54	0.50	0.68	0.52	0.51
	(0.47)	(0.38)	(0.50)	(0.42)	(0.50)	(0.50)	(0.47)	(0.50)	(0.50)
Economy first	0.14	0.05	0.13	0.09	0.15	0.14	0.06	0.09	0.16
	(0.35)	(0.22)	(0.34)	(0.29)	(0.35)	(0.35)	(0.24)	(0.28)	(0.37)
