# Supplementary material for: In science we (should) trust: Expectations and compliance across nine countries during the COVID-19 pandemic
Source: PLoS One. 2021 Jun 4;16(6):e0252892. doi: 10.1371/journal.pone.0252892 (PMC8177647; doi:10.1371/journal.pone.0252892)
Supplement: S2 Table — All measures are on a 1–4 scale, where 1 is the lowest level of trust and 4 is the highest, except the Trust question that is used in the World Values Survey, which takes a value of 1 if people say they can generally trust others and 0 if not. (PDF) [file pone.0252892.s002.pdf]

S2 Table. Trust – Country level

	CH	CO	GE	IT	MX	SK	SP	UK	US
Trust WVS	0.61	0.07	0.41	0.16	0.08	0.30	0.33	0.48	0.36
	(0.49)	(0.25)	(0.49)	(0.36)	(0.27)	(0.46)	(0.47)	(0.49)	(0.48)
Family	3.73	3.51	3.68	3.69	3.29	3.53	3.71	3.50	3.23
	(0.54)	(0.83)	(0.74)	(0.72)	(1.01)	(0.72)	(0.73)	(0.88)	(1.03)
Neighbors	3.02	2.40	2.84	2.77	2.25	2.81	2.85	2.78	2.62
	(0.64)	(0.83)	(0.80)	(0.74)	(0.83)	(0.67)	(0.80)	(0.80)	(0.89)
Juniors	2.78	2.09	2.42	2.44	2.11	2.32	2.58	2.36	2.35
	(0.75)	(0.78)	(0.76)	(0.79)	(0.82)	(0.73)	(0.87)	(0.75)	(0.88)
Seniors	2.77	2.75	2.78	2.67	2.70	2.47	2.89	2.85	2.78
	(0.74)	(0.97)	(0.76)	(0.85)	(1.01)	(0.76)	(0.96)	(0.83)	(0.95)
Doctors	3.48	3.30	3.18	3.51	3.13	3.18	3.62	3.26	3.04
	(0.69)	(0.89)	(0.80)	(0.78)	(1.03)	(0.79)	(0.77)	(0.89)	(1.00)
Scientists	3.38	3.23	3.06	3.30	3.07	3.04	3.51	3.08	2.98
	(0.68)	(0.89)	(0.83)	(0.83)	(1.04)	(0.78)	(0.80)	(0.88)	(1.00)
WHO	3.29	2.93	2.85	2.97	2.96	2.49	3.02	2.91	2.74
	(0.74)	(0.95)	(0.88)	(0.93)	(1.05)	(0.90)	(0.93)	(0.91)	(1.09)
Government	3.42	2.11	2.72	2.56	2.04	2.65	2.09	2.48	2.30
	(0.75)	(0.99)	(0.93)	(0.97)	(0.95)	(0.88)	(1.01)	(0.92)	(0.98)

All measures are on a 1-4 scale, where 1 is the lowest level of trust and 4 is the highest, except the Trust question that is used in the World Values Survey, which takes a value of 1 if people say they can generally trust others and 0 if not.
