# Supplementary material for: In science we (should) trust: Expectations and compliance across nine countries during the COVID-19 pandemic
Source: PLoS One. 2021 Jun 4;16(6):e0252892. doi: 10.1371/journal.pone.0252892 (PMC8177647; doi:10.1371/journal.pone.0252892)
Supplement: S3 Table — (PDF) [file pone.0252892.s003.pdf]

S3 Table. Pairwise correlations between trust in different groups

	Family	Neighbors	Juniors	Seniors	Government	Police	Doctors	Science
Neighbors	0.563							
Juniors	0.418	0.606						
Seniors	0.484	0.549	0.486					
Government	0.342	0.410	0.365	0.347				
Police	0.470	0.506	0.409	0.439	0.599			
Doctors	0.622	0.487	0.388	0.485	0.436	0.609		
Science	0.556	0.452	0.373	0.465	0.431	0.551	0.770	
WHO	0.431	0.370	0.356	0.401	0.451	0.482	0.604	0.669

All pairwise correlations significant at the 1% level
