# Supplementary material for: In science we (should) trust: Expectations and compliance across nine countries during the COVID-19 pandemic
Source: PLoS One. 2021 Jun 4;16(6):e0252892. doi: 10.1371/journal.pone.0252892 (PMC8177647; doi:10.1371/journal.pone.0252892)
Supplement: S5 Table — (PDF) [file pone.0252892.s005.pdf]

S5 Table. After the lockdown – Country level

	CH	CO	GE	IT	MX	SK	SP	UK	US
Behavior									
Social distancing	0.97	0.99	0.94	0.99	0.94	0.96	0.99	0.98	0.94
	(0.17)	(0.11)	(0.24)	(0.08)	(0.25)	(0.20)	(0.08)	(0.15)	(0.23)
Stay home	0.96	0.97	0.87	0.99	0.92	0.93	0.99	0.97	0.92
	(0.19)	(0.17)	(0.34)	(0.09)	(0.27)	(0.26)	(0.11)	(0.16)	(0.27)
Normative belief									
Social distancing	0.97	0.99	0.91	0.98	0.96	0.95	0.99	0.97	0.92
	(0.17)	(0.10)	(0.29)	(0.12)	(0.19)	(0.21)	(0.12)	(0.17)	(0.27)
Stay home	0.95	0.98	0.85	0.97	0.95	0.94	0.98	0.95	0.91
	(0.21)	(0.14)	(0.36)	(0.16)	(0.22)	(0.25)	(0.14)	(0.21)	(0.29)
Empirical expectations									
Social distancing	80.27	68.54	70.25	70.84	49.98	68.89	82.18	75.90	67.75
	(20.24)	(26.32)	(18.38)	(17.69)	(20.53)	(23.46)	(18.77)	(19.57)	(21.98)
Stay home	80.97	69.76	66.76	70.91	49.12	68.09	81.75	74.25	66.63
	(20.34)	(27.21)	(19.22)	(17.00)	(20.39)	(23.81)	(18.53)	(19.15)	(22.10)
Normative expectations									
Social distancing	81.16	70.99	70.65	73.03	53.87	70.30	82.95	76.82	69.30
	(20.15)	(26.76)	(19.25)	(17.72)	(21.78)	(23.59)	(18.92)	(19.66)	(22.17)
Stay home	81.69	71.26	66.94	72.21	54.59	68.10	82.29	76.02	67.82
	(20.01	(27.02)	(19.11)	(18.16)	(22.60)	(24.42)	(19.38)	(19.61)	(22.56)
