# Supplementary material for: In science we (should) trust: Expectations and compliance across nine countries during the COVID-19 pandemic
Source: PLoS One. 2021 Jun 4;16(6):e0252892. doi: 10.1371/journal.pone.0252892 (PMC8177647; doi:10.1371/journal.pone.0252892)
Supplement: S6 Table — (PDF) [file pone.0252892.s006.pdf]

S6 Table. Experimental outcomes – Country level

		High/High	High/Low	Low/High	Low/Low
China	Stay home	7.54	6.36	6.78	5.71
		(2.59)	(2.86)	(2.60)	(3.05)
	Social distancing	7.49	6.38	6.80	5.61
		(2.65)	(2.80)	(2.59)	(2.98)
Colombia	Stay home	7.88	6.16	5.80	5.02
		(2.14)	(2.64)	(2.82)	(2.87)
	Social distancing	7.88	6.29	5.73	4.74
		(2.27)	(2.61)	(2.90)	(2.85)
Germany	Stay home	7.53	5.74	6.08	4.28
		(1.83)	(2.31)	(2.23)	(2.64)
	Social distancing	7.67	5.73	6.29	4.39
		(1.83)	(2.31)	(2.18)	(2.62)
Italy	Stay home	7.62	5.98	5.62	4.72
		(1.94)	(2.46)	(2.46)	(2.74)
	Social distancing	7.57	5.99	5.57	4.70
		(2.01)	(2.40)	(2.49)	(2.68)
Mexico	Stay home	6.67	5.44	4.99	5.02
		(2.74)	(2.68)	(2.75)	(2.85)
	Social distancing	7.00	5.48	4.94	4.99
		(2.70)	(2.67)	(2.77)	(2.95)
South Korea	Stay home	6.63	6.29	4.98	5.09
		(2.72)	(2.94)	(2.75)	(2.97)
	Social distancing	6.63	6.47	5.09	5.16
		(2.81)	(2.99)	(2.77)	(2.97)
Spain	Stay home	8.50	6.01	5.77	4.63
		(1.65)	(2.57)	(2.56)	(2.89)
	Social distancing	8.53	5.93	5.69	4.57
		(1.69)	(2.47)	(2.57)	(2.96)
United Kingdom	Stay home	8.21	6.36	6.16	4.95
		(1.94)	(2.50)	(2.57)	(3.01)
	Social distancing	8.22	6.30	6.17	4.91
		(1.98)	(2.49)	(2.58)	(2.90)
United States	Stay home	7.58	6.18	5.71	4.92
		(2.53)	(2.87)	(2.98)	(3.27)
	Social distancing	7.66	6.15	5.74	4.86
		(2.53)	(2.92)	(3.03)	(3.31)
