# Supplementary material for: In science we (should) trust: Expectations and compliance across nine countries during the COVID-19 pandemic
Source: PLoS One. 2021 Jun 4;16(6):e0252892. doi: 10.1371/journal.pone.0252892 (PMC8177647; doi:10.1371/journal.pone.0252892)
Supplement: S7 Table — Standard errors in parentheses, ** p<0.01, * p<0.05. OLS estimates with individual (gender, age, education and location) and country controls. Low-Low treatment used as the benchmark (PDF) [file pone.0252892.s007.pdf]

S7 Table. Compliance likelihood (SD and SH) and expectations

	(1)<br>Social distance	(2)<br>Stay at home
High Normative-<br>High Empirical	2.747**	2.647**
	(0.071)	(0.071)
High Normative-<br>Low Empirical	1.200**	1.132**
	(0.072)	(0.071)
Low Normative-<br>High Empirical	0.905**	0.847**
	(0.071)	(0.071)
Constant	4.686**	4.887**
	(0.194)	(0.193)
Individual controls	Yes	Yes
Country controls	Yes	Yes
Observations	10,986	10,986
R-squared	0.134	0.131

Standard errors in parentheses, ** p<0.01, * p<0.05
OLS estimates with individual (gender, age, education and location) and country controls. Low-Low treatment used as the benchmark
