# Supplementary material for: In science we (should) trust: Expectations and compliance across nine countries during the COVID-19 pandemic
Source: PLoS One. 2021 Jun 4;16(6):e0252892. doi: 10.1371/journal.pone.0252892 (PMC8177647; doi:10.1371/journal.pone.0252892)
Supplement: S8 Table — Standard errors in parentheses, ** p<0.01, * p<0.05. OLS estimates with individual (gender, age, education and location) and country controls. Low-Low treatment used as the benchmark. (PDF) [file pone.0252892.s008.pdf]

S8 Table. Trust in Science and Government (High), expectations, and compliance likelihood (SD and SH)

	Science		Government	
	(1)	(2)	(3)	(4)
	Social distance	Stay at home	Social distance	Stay at home
Trust (High)	0.169	0.107	0.410**	0.466**
	(0.159)	(0.159)	(0.104)	(0.103)
High N-High E	1.701**	1.607**	2.834**	2.746**
	(0.209)	(0.208)	(0.107)	(0.107)
High N-High E*Trust	1.187**	1.181**	-0.159	-0.181
	(0.222)	(0.222)	(0.144)	(0.143)
High N-Low E	0.958**	0.991**	1.193**	1.154**
	(0.205)	(0.204)	(0.108)	(0.107)
High N-Low E*Trust	0.281	0.163	0.00634	-0.0469
	(0.219)	(0.218)	(0.144)	(0.143)
Low N –High E	0.512*	0.493*	0.770**	0.720**
	(0.209)	(0.209)	(0.107)	(0.106)
Low N-High E *Trust	0.446*	0.401	0.252	0.237
	(0.223)	(0.222)	(0.144)	(0.143)
Constant	4.642**	4.880**	4.400**	4.564**
	(0.236)	(0.235)	(0.203)	(0.202)
Individual controls	Yes	Yes	Yes	Yes
Country controls	Yes	Yes	Yes	Yes
Observations	10,986	10,986	10,986	10,986
R-squared	0.142	0.138	0.140	0.138

In columns 1 and 2 trust (high) refers to having high trust in science; in columns 3 and 4 it refers to having high trust in government. Standard errors in parentheses, ** p<0.01, * p<0.05. OLS estimates with individual (gender, age, education and location) and country controls. Low-Low treatment used as the benchmark.
