# Supplementary material for: In science we (should) trust: Expectations and compliance across nine countries during the COVID-19 pandemic
Source: PLoS One. 2021 Jun 4;16(6):e0252892. doi: 10.1371/journal.pone.0252892 (PMC8177647; doi:10.1371/journal.pone.0252892)
Supplement: S9 Table — In columns 1 and 2 trust (1–4) refers to the level of trust in science; in columns 3 and 4 it refers to having the level of trust in government. Standard errors in parentheses, ** p<0.01, * p<0.05. OLS estimates with individual (gender, age, education and location) and country controls. Low-Low treatment used as the benchmark. (PDF) [file pone.0252892.s009.pdf]

S9 Table. Trust in Science and in Government (1-4 scale), expectations, and compliance likelihood (SD and SH)

	Science		Government	
	(1)	(2)	(3)	(4)
	Social distance	Stay at home	Social distance	Stay at home
Trust (1-4)	0.143*	0.136	-0.235**	-0.245**
	(0.0699)	(0.070)	(0.0530)	(0.0527)
High N-High E	3.698**	3.506**	2.544**	2.531**
	(0.179)	(0.179)	(0.192)	(0.191)
High N-High E*Trust	0.561**	0.504**	0.0818	0.0480
	(0.0977)	(0.097)	(0.0730)	(0.0726)
High N-Low E	1.438**	1.305**	1.241**	1.131**
	(0.178)	(0.178)	(0.193)	(0.192)
High N-Low E*Trust	0.132	0.0928	-0.0159	0.00229
	(0.0964)	(0.096)	(0.0732)	(0.0728)
Low N-High E	1.162**	1.045**	1.309**	1.302**
	(0.180)	(0.179)	(0.192)	(0.191)
Low N-High E*Trust	0.147	0.112	-0.165*	-0.186*
	(0.0976)	(0.097)	(0.0727)	(0.0724)
Constant	5.079**	5.242**	5.130**	5.334**
	(0.230)	(0.229)	(0.230)	(0.229)
Individual controls	Yes	Yes	Yes	Yes
Country controls	Yes	Yes	Yes	Yes
Observations	10,986	10,986	10,986	10,986
R-squared	0.145	0.141	0.142	0.141

In columns 1 and 2 trust (1-4) refers to the level of trust in science; in columns 3 and 4 it refers to having the level of trust in government. Standard errors in parentheses, ** $p<0.01$, * $p<0.05$. OLS estimates with individual (gender, age, education and location) and country controls. Low-Low treatment used as the benchmark.
