# Supplementary material for: In science we (should) trust: Expectations and compliance across nine countries during the COVID-19 pandemic
Source: PLoS One. 2021 Jun 4;16(6):e0252892. doi: 10.1371/journal.pone.0252892 (PMC8177647; doi:10.1371/journal.pone.0252892)
Supplement: S10 Table — Standard errors in parentheses, ** p<0.01, * p<0.05. OLS estimates with individual (gender, age, education and location) and country controls. (PDF) [file pone.0252892.s010.pdf]

S10 Table. Trust in Government and Science (High) interaction by different levels of expectations and compliance likelihood (SD)

	(1)<br>High-High	(2)<br>High-Low	(3)<br>Low-High	(4)<br>Low-Low
Trust in Gov (H)	0.292	0.488	0.600	0.308
	(0.295)	(0.344)	(0.348)	(0.387)
Trust in Science (H)	1.140**	0.385*	0.500**	0.205
	(0.160)	(0.182)	(0.190)	(0.213)
H*H	-0.114	-0.140	-0.195	0.0488
	(0.308)	(0.361)	(0.366)	(0.404)
Constant	3.518**	5.021**	5.982**	6.336**
	(0.346)	(0.389)	(0.409)	(0.445)
Individual controls	Yes	Yes	Yes	Yes
Country controls	Yes	Yes	Yes	Yes
Observations	2,749	2,750	2,741	2,746
R-squared	0.117	0.030	0.053	0.025

Standard errors in parentheses, ** $p<0.01$, * $p<0.05$. OLS estimates with individual (gender, age, education and location) and country controls. High-High refers to both normative and empirical expectations being high; High-Low refers to High normative expectations and Low empirical expectations; Low-High refers to Low normative expectations and high empirical expectations; and Low-Low refers to both normative and empirical expectations being low.
