# Supplementary material for: In science we (should) trust: Expectations and compliance across nine countries during the COVID-19 pandemic
Source: PLoS One. 2021 Jun 4;16(6):e0252892. doi: 10.1371/journal.pone.0252892 (PMC8177647; doi:10.1371/journal.pone.0252892)
Supplement: S11 Table — Probit estimations with individual (gender, age, education and location) and country controls, figures are marginal effects, relative to the benchmark (low expectations and low level of trust), using self-reported compliance before the lockdown as dependent variable. (PDF) [file pone.0252892.s011.pdf]

S11 Table. Probit results for self-reported compliance

	(1)<br>Science	(2)<br>Government
Low Trust, Low Expectations	*0.6506*	*0.6493*
High trust (Low expectations)	-0.0439***	-0.0283**
	(0.0155)	(0.0115)
Predicted	*.6067*	*.6210*
(Low trust) High expectations	0.0218	0.172***
	(0.0235)	(0.0133)
Predicted	*.6724*	*.8213*
High trust * High expectations	0.278***	0.164***
	(0.0205)	(0.0172)
Predicted	*.9286*	*.8133*
Low Expectations: Δ Trust	*-4.39pp****	*-2.83pp***
	(-7%)	*(-4%)*
High Expectations: Δ Trust	*+25.62pp****	*-0.80pp*
	(+38%)	*(-1%)*
Low Trust: Δ Expectations	*+2.18pp*	*+17.2pp****
	(+3%)	*(+26%)*
High Trust: Δ Expectations	*+32.19pp****	*+19.23pp****
	(+53%)	*(+31%)*
Individual controls	Yes	Yes
Country controls	Yes	Yes
Observations	7,901	7,901

Standard errors in parentheses, *** p<0.01, ** p<0.05, * p<0.1

Probit estimations with individual (gender, age, education and location) and country controls, figures are marginal effects, relative to the benchmark (low expectations and low level of trust), using self-reported compliance before the lockdown as dependent variable.
