# Supplementary figures and images for: In science we (should) trust: Expectations and compliance across nine countries during the COVID-19 pandemic
Source: PLoS One. 2021 Jun 4;16(6):e0252892. doi: 10.1371/journal.pone.0252892 (PMC8177647; doi:10.1371/journal.pone.0252892)

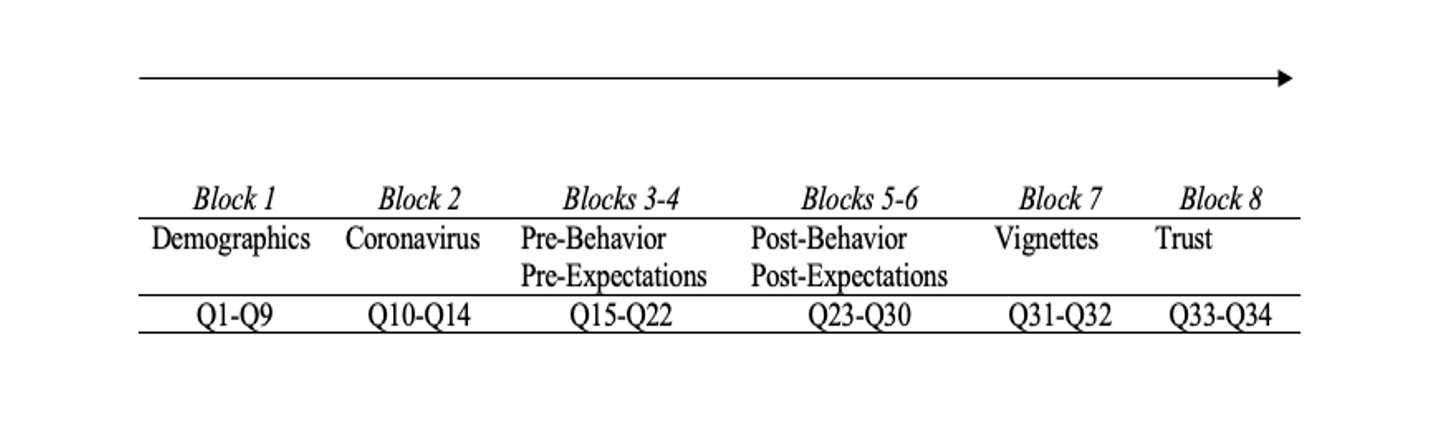

Supplement: S1 Fig — (TIF) [file pone.0252892.s012.tif]

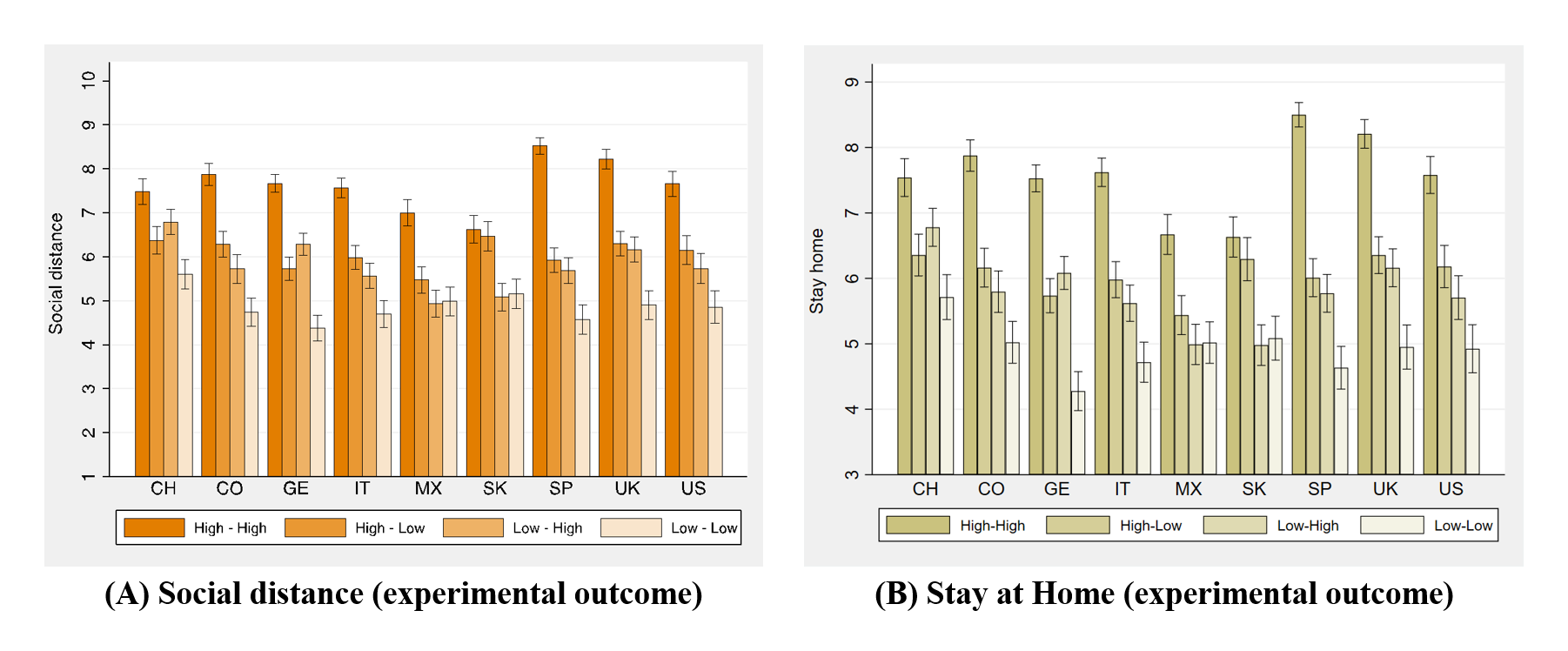

Supplement: S2 Fig — (TIF) [file pone.0252892.s013.tif]

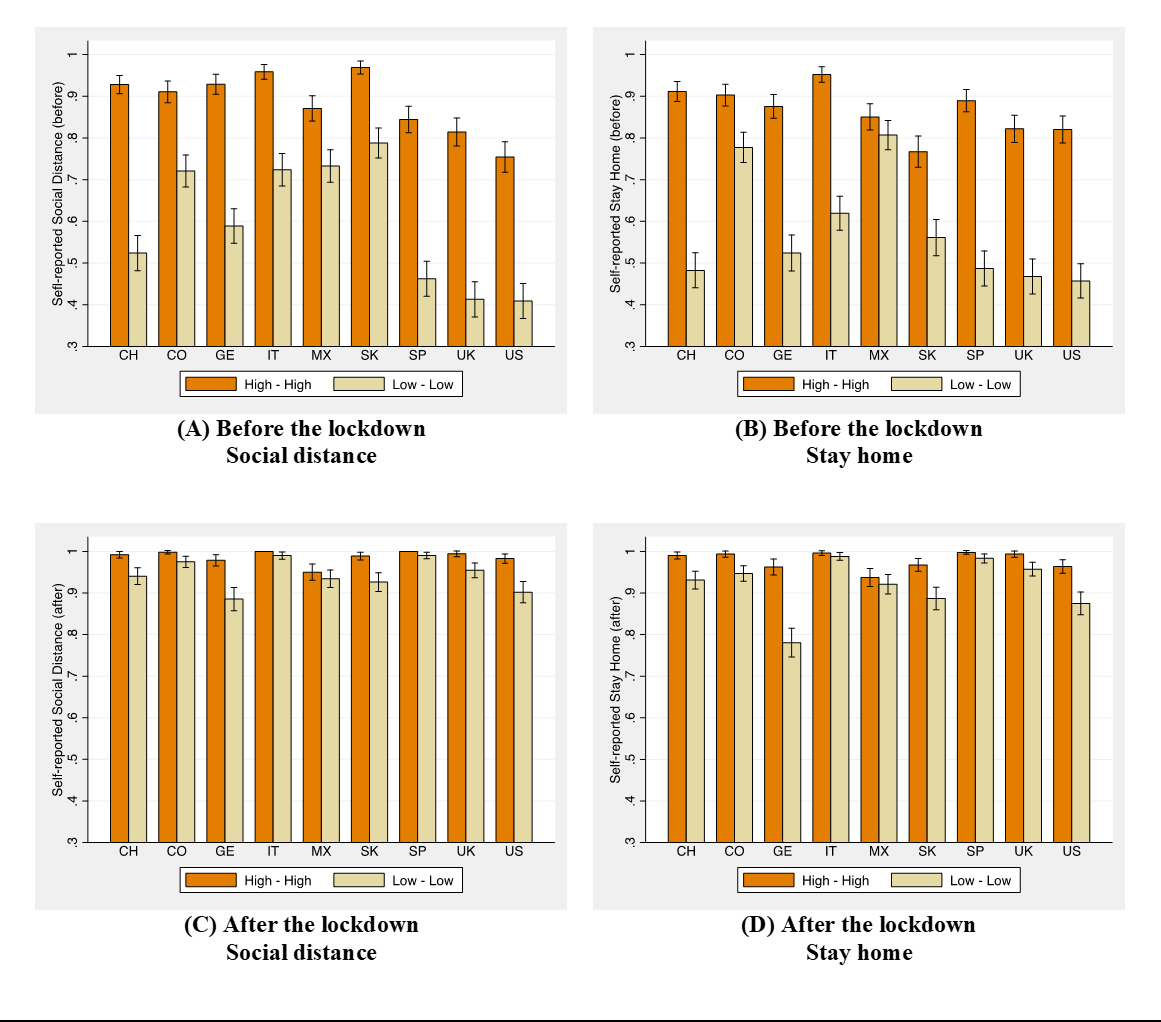

Supplement: S3 Fig — Per the survey, not including incongruent expectations. (TIF) [file pone.0252892.s014.tif]
